# Neuronal-Derived EV Biomarkers Track Cognitive Decline in Alzheimer’s Disease

**DOI:** 10.3390/cells11030436

**Published:** 2022-01-27

**Authors:** Erden Eren, Jeannie-Marie Leoutsakos, Juan Troncoso, Constantine G. Lyketsos, Esther S. Oh, Dimitrios Kapogiannis

**Affiliations:** 1 Intramural Research Program, Laboratory of Clinical Investigation, National Institute on Aging, Baltimore, MD 21224, USA; erden.eren@nih.gov; 2 Department of Psychiatry and Behavioral Sciences, Johns Hopkins School of Medicine, Baltimore, MD 21205, USA; jeannie-marie@jhu.edu (J.-M.L.); kostas@jhmi.edu (C.G.L.); 3 Department of Medicine, Division of Geriatric Medicine and Gerontology, Johns Hopkins School of Medicine, Baltimore, MD 21287, USA; 4 Department of Neurology, Johns Hopkins School of Medicine, Baltimore, MD 21224, USA; troncoso@jhmi.edu; 5 Richman Family Precision Medicine Center of Excellence in Alzheimer’s Disease, Johns Hopkins School of Medicine, Baltimore, MD 21224, USA

**Keywords:** extracellular vesicles, autopsy, Alzheimer’s disease, mixed pathology, exosomes, neuron-derived extracellular vesicles

## Abstract

The hallmarks of Alzheimer’s disease (AD) pathology are senile plaques containing amyloid-beta (Aβ) and neurofibrillary tangles containing hyperphosphorylated tau. Additional pathologies often co-exist, whereas multiple pathogenic mechanisms are involved in AD, especially synaptic degeneration, which necessitate the need for synaptic integrity-related biomarkers alongside Aβ- and tau-related biomarkers. Plasma neuron-derived Extracellular Vesicles EVs (NDEVs) provide biomarkers related to Aβ and tau and synaptic degeneration. Here, to further establish the latter as a “liquid biopsy” for AD, we examined their relationship with ante-mortem cognition in pathologically-confirmed AD cases. We immunoprecipitated NDEVs by targeting neuronal marker L1CAM from ante-mortem plasma samples from 61 autopsy-confirmed cases of pure AD or AD with additional pathologies and measured Aβ_42_, p181-Tau, total Tau, synaptophysin, synaptopodin and three canonical EV markers, CD63, CD81 and CD9. Higher NDEV Aβ_42_ levels were consistently associated with better cognitive status, memory, fluency, working memory and executive function. Higher levels of NDEV synaptic integrity-related biomarkers were associated with better performance on executive function tasks. Our findings motivate the hypothesis that releasing Aβ_42_-laden NDEVs may be an adaptive mechanism in AD.

## 1. Introduction

The pathogenesis of Alzheimer’s disease (AD) involves the progressive accumulation of extracellular plaques containing misfolded amyloid-beta (Aβ) and intracellular neurofibrillary tangles (NFT) containing misfolded Tau, which lead to synaptic loss and neurodegeneration. The definitive diagnosis of AD still relies on demonstrating the presence of these hallmark pathologies during autopsy [1,2]. However, it is increasingly being recognized that AD pathologic changes are rarely the only ones to be found in individuals who died with Mild Cognitive Impairment (MCI) or AD dementia. Additional pathologies, such as cerebrovascular pathologies of variable severity (from frank infarcts to microscopic cortical infarcts to microvascular disease), cortical and subcortical Lewy bodies, TDP-43 inclusions, and lesions associated with Frontotemporal Lobar Degenerations (FTLD) often co-exist with canonical AD pathologic lesions [3]. However precise they may be, neuropathological assessments are not useful when it comes to diagnosing a living individual with suspected AD in the clinic or establishing eligibility for clinical trials.

To accurately diagnose living individuals with AD, detect additional pathologies and predict the course of their disease, we need to rely on biomarkers. The field has experienced a paradigm shift so that the clinical diagnosis of probable AD is currently supplemented by a diagnosis of high, medium or low probability of underlying AD pathology based on biomarkers for “A/T/N”. This is a flexible and expandable conceptual framework with the dual goal of categorizing various AD biomarkers that have been developed over the years and staging the disease based on biomarkers that reflect different pathologic processes [4]. Specifically, “A” reflects amyloid pathologies and is currently best being ascertained by CSF Aβ_42_ and/or amyloid PET; “T” refers to fibrillar tau and is currently being assessed by CSF p-Tau and/or Tau PET; and “N” denotes neurodegeneration or neuronal injury and is currently being assessed by CSF total-Tau and atrophy on structural MRI [4]. Furthermore, biomarkers reflecting synaptic loss, an early feature of AD that correlates with cognitive impairment [5], are often being considered as part of “N”. Both IWG and NIA-AA concluded that the minimum requirement for an individual to belong in the AD spectrum is a demonstration of abnormal amyloid-related biomarkers [6,7]; therefore, currently, biomarkers reflecting “A” are foundational.

Current A/T/N biomarkers provide greater sensitivity and specificity compared to clinical diagnosis and cognitive tests [8], yet they depend on CSF sampling and/or amyloid and/or Tau PET, which are invasive and/or expensive and not widely available. Blood-based biomarkers are without doubt less invasive, more convenient, affordable and widely available compared to CSF and PET biomarkers. Moreover, current A/T/N biomarkers have significant limitations, most notably the fact that they reach a plateau during the progression of clinical AD, which makes them less than ideal for monitoring disease evolution and assessment of treatment response [9,10].

Extracellular vesicles (EVs) are membranous nanoparticles released from all cells including neurons, ranging from 30 nm to 1 µm in diameter and originating from the endosomal system (exosomes) or through direct shedding from the plasma membrane (microvesicles) [11,12]. It is widely accepted that their cargo reflects the physiologic state and pathogenic processes in the originating cell [12]. We and others have contributed to the development of a new class of blood biomarkers for AD by leveraging the cargo of neuron-derived extracellular vesicles (NDEVs) selectively immunocaptured from plasma [13,14,15,16]. Previous studies have shown that NDEV biomarkers, including Aβ_42_, p-Tau and total Tau, can accurately discriminate between individuals with clinical AD and controls, predict future AD diagnosis at the preclinical stage, and predict conversion from MCI to AD dementia and future cognitive decline among individuals at higher genetic risk for the disease [13,15,17]. Moreover, others have shown that NDEV Aβ_42_ and Tau levels are strongly correlated with their CSF levels [17]. Furthermore, synaptic proteins in NDEVs have shown significant correlations with cross-sectional Mini-Mental State Examination (MMSE) scores, suggesting that they may be able to reflect disease severity spanning clinical disease [18]. Conceptually, amyloid, Tau and synaptic integrity-related NDEV biomarkers can be readily incorporated into the A/T/N classification scheme. However, their ability to discriminate between individuals harboring additional pathologies and those with pure AD, as well as to track pathologic progression and cognitive decline in individuals with clinical AD, has not been explored.

In this study, we hypothesized that NDEV biomarkers for A/T/N may achieve these two goals: discriminate between individuals with definitive AD diagnosis with or without additional pathologies and track cognitive decline across a wide range of disease severity. To that end, we analyzed plasma samples of 61 cases from the Johns Hopkins Alzheimer’s disease Research Center (ADRC) who received an ante-mortem dementia diagnosis and underwent autopsy. We found that the NDEV markers examined were not able to discriminate between individuals with Pure AD (PurAD) pathology and those with AD and mixed pathologies (mild or severe). However, NDEV Aβ_42_ levels were associated with cognitive performance across multiple cognitive domains (and a trend with Braak stage), whereas NDEV biomarkers for “T” (p181-Tau) and “N” (t-Tau and synaptophysin and synaptopodin) showed associations with ante-mortem cognitive performance.

## 2. Materials and Methods

### 2.1. Participants

Participants were 61 volunteers with the Johns Hopkins Alzheimer’s Disease Research Center (ADRC) Clinical Core for whom cognitive assessments, ante-mortem plasma, and neuropathological data were available and who had AD pathology present at autopsy. A Johns Hopkins University Institutional Review Board oversaw the activities of the ADRC Clinical Core. Written informed consent from each participant was obtained, as well as post-mortem autopsy consent provided by a legally authorized representative.

### 2.2. Neuropathological and Cognitive Assessments 

The ADRC Clinical Core recruits and follows diverse participants aged 60 and older and/or who suffer from cognitive disorders. The ADRC has been following participants for over 30 years and offers a brain donation (autopsy) option. As of 31 December 2020, the ADRC had 493 active participants. 

Participants are seen annually for in-person comprehensive assessments involving standardized history, neuropsychological testing, as well as neurologic and mental status examinations. At the core of this annual assessment is the Uniform Data Set (UDS; https://naccdata.org/data-collection/forms-documentation/uds-3) developed by the NIA-funded AD Centers program, kept in a database curated by the National Alzheimer’s Coordinating Center (NACC; https://naccdata.org). Blood specimens (i.e., plasma, serum and DNA), for the purposes of studying blood biomarkers, were collected from approximately 96% of participants over the last 14 years. 

Approximately 75% of participants consented to brain donation and approximately 80% of individuals who died while enrolled in this program received an autopsy in the department of pathology at Johns Hopkins. Standardized pathologic examination of the brain was conducted for diagnostic purposes by the ADRC Neuropathology Core and recorded in the NACC database. This autopsy case series involved all ADRC participants who came to autopsy over the last 14 years for whom stored plasma was available.

Cognitive assessments [19] including Logical Memory, Digit Span Forward, Digit Span Backwards, WAIS-R Digit Symbol, Logical Memory delayed, animal fluency, vegetable fluency, and Trails A and B were conducted at ADRC visits after 2005 when NACC switched from the minimum dataset (MDS) [20] to the more comprehensive uniform dataset (UDS) [21]. Visits prior to 2015 included MMSE, and visits after 2015 included the Montreal Cognitive Assessment MoCA [22]. To enable comparison across participants and time points we used a published “crosswalk” to translate MOCA scores to estimated MMSE scores [23]. 

### 2.3. Extracellular Vesicle Isolation

Neuron-derived extracellular vesicles were isolated from 0.5 mL human plasma samples with minor modifications to our previously published protocol [14]. Briefly, aliquots of 0.5 mL plasma were incubated with 5 µL of Thrombin preparation (System Biosciences, Mountain View, CA, USA) for 30 min at room temperature, followed by addition of 0.495 mL of calcium- and magnesium-free Dulbecco’s balanced salt solution (DBS) with protease inhibitor cocktail (Roche, Indianapolis, IN, USA) and phosphatase inhibitor cocktail (Thermo Fisher Scientific; DBS++) [16,24]. After centrifugation at 4000× *g* for 20 min at 4 °C, total particles were precipitated with 252 μL per tube of ExoQuick (System Biosciences, Mountain View, CA, USA) and centrifugation at 1500× *g* for 20 min at 4 °C. Of note, recent investigations have shown that ExoQuick recovers the highest concentration of EV material from small amounts of plasma among competing methods [25], which is the main objective of this step. To isolate an enriched subpopulation of NDEVs with high purity, total particles (TEVs) were resuspended in 0.7 mL of ddH_2_O supplemented with protease and phosphatase inhibitors and 0.5 mL of TEVs were incubated for 60 min at room temperature with 2.7 μg of mouse anti-human CD171 (L1CAM neural adhesion protein) biotinylated antibody (clone 5G3; eBiosciences, San Diego, CA, USA) in 3% BSA with mixing for 120 min, followed by addition of 26 μL of streptavidin agarose Ultralink resin, respectively (ThermoFisher Scientific, Waltham, MA, USA) in 3% BSA and incubation for 60 min at room temperature with mixing. After centrifugation at 800× *g* for 10 min at 4 °C and removal of the supernatant, each pellet was suspended in cold 0.1 M glycine-HCl (pH 3.0) by gentle mixing for 10 s and centrifuged at 4500× *g* for 5 min, all at 4 °C. Supernatants then were transferred to clean tubes containing 3% BSA and 1 M Tris-HCl (pH 8.0) and mixed gently. An aliquot of 10 μL of intact NDEVs was removed from each tube for NTA analysis before addition of mammalian protein extraction reagent (M-PER, ThermoFisher Scientific Waltham, MA, USA). Resultant lysates of NDEVs were stored at −80 °C.

**Figure 1:**
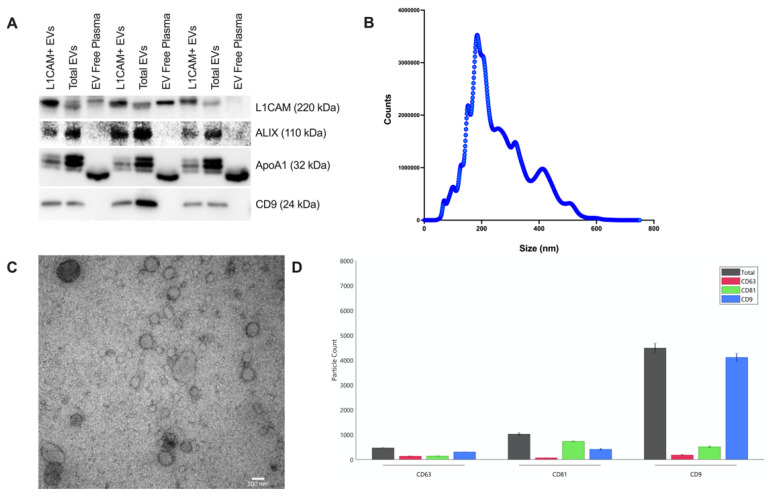
Western blot analysis of L1CAM, Alix, ApoA1 and CD9 in L1CAM + NDEVs, Total EVs, and EV free plasma, *N* = 3 cognitively normal individuals (**A**). Representative size distribution of three NDEV samples by NTA analysis. Average number of NDEVs was 2.73 × 10^10^ ± 1.72 × 10^10^ particles/mL and the mode of NDEV diameters was 179.44 nm ± 51.59 nm (**B**). Representative negative stain (Oxalate) Transmission Electron Microscopy images of NDEV samples (**C**). Exoview analysis of NDEVs by using ExoView Tetraspanin chip and characteristic image; the analysis demonstrates that NDEVs express all major tetraspanins and that a majority of them express CD9 compared to CD81 and CD63 (**D**).

We performed NDEV characterization by Western blots (more extensive NDEV characterization was reported as part of previous manuscripts [15,16]), Exoview analysis and Transmission Electron Microscopy (TEM) imaging. The protein concentration of the samples was measured with BCA assay according to the manufacturer’s protocol. Five micrograms of each sample were mixed with LDS sample loading buffer (ThermoFisher Scientific, Waltham, MA, USA) and boiled at 70 °C for 10 min. Samples were separated in 4–12% NuPAGE^TM^ Bis-Tris 1.0 mm gel system (ThermoFisher Scientific, Waltham, MA, USA) and EV proteins were transferred system onto nitrocellulose membrane by iBlot 2 transfer (ThermoFisher Scientific, Waltham, MA, USA). Membranes were blocked with 5% non-fat dried milk (Cell Signaling, Denver, MA, USA, 9999) in 1X TBS-T for 1 hr at RT followed by overnight incubation at +4 °C with following antibodies; CD9 (1:500; Biolegend, San Diego, CA, USA, 312102) and ALIX (1:1000; Cell Signaling, Denver, MA, USA, 2171), as positive EV markers; L1CAM (1:1000; BD Biosciences, SanDiego, CA, USA, 554273), the target of enrichment; and ApoA1 (1:500, R&D Systems, Minneapolis, MN, USA; AF3664), to examine relative plasma purity of lipoprotein contaminants. After incubation with primary antibodies, membranes were incubated with HRP-conjugated anti-mouse (1:3000; Cell Signaling, Denver, MA, USA, 7076) for CD9 and ALIX and L1CAM and anti-goat (1:5000; Santacruz, Santa Cruz, CA, USA) for ApoA1. After washing four times with 1X TBS-T, membranes were incubated with Amersham ECL prime solution (GE Healthcare, Silver Spring, MD, USA) and images were captured with Azure Sapphire Biomolecular imaging system (Azure Biosystems, Dublin, CA, USA) (Figure 1A). In addition, the average diameter and concentration of intact NDEVs were quantified by Nanoparticle Tracking Analysis (NTA) with NanoSight NS500 (Malvern Panalytical Ltd., Worcestershire, UK) (Figure 1B).

For TEM, NDEV samples were fixed with 2% PFA and samples were deposited on Formvar-carbon coated EM grids for 20 min. Grids were then washed with PBS and incubated with 1% glutaraldehyde for 5 min. Afterward, grids were washed with DI water eight times. Grids were treated with uranyl–oxalate solution for 5 min and incubated with methyl cellulose-UA for 10 min on ice. After removing any excess liquid on grids, they were air-dried. TEM images were acquired with Hitachi 7600 TEM (Figure 1C). 

We analyzed tetraspanin expression in NDEVs by ExoView R200 (Nanoview Biosciences, Brighton, MA, USA) following the manufacturer’s instructions. Briefly, NDEV samples were incubated on the ExoView Tetraspanin chip on a 24-well plate overnight (for 16 h); then, the chip was washed three times with 1X Solution A; finally, we added detection antibodies anti-CD81(CF 555), anti-CD63 (CF 488) and anti-CD9 (CF 647) for one hour. The chip was then washed twice with solution A, once with solution B, and finally with DI water. Images were acquired and analyzed with ExoScan software (Nanoview Biosciences, Brighton, MA, USA) (Figure 1D).

### 2.4. Biomarker Measurements

We quantified Aβ_42_, p181-Tau and total Tau levels using a Luminex-based multiplex assay (EMD Millipore Corporation, Billerica, MA, USA), and used ELISAs to quantify the presynaptic marker synaptophysin (Cusabio Biotech Co., Ltd., Houston, TX, USA) and the post-synaptic marker synaptopodin (LifeSpan BioSciences, Seattle, WA, USA) in NDEVs. Furthermore, we quantified three tetraspanin molecules (CD63, CD81 and CD9) for NDEVs, which are widely considered as canonical EV markers [26], using a novel electrochemiluminescence-based multiplex immunoassay for intact EVs developed by Meso Scale Discovery (Meso Scale Discovery, Rockville, MD, USA). This assay depends on capturing antibodies against three individual tetraspanins, CD63, CD9, CD81 and isotype control mouse IgG and cocktail of all three tetraspanin antibodies conjugated with SULFO-TAG for detection. The geometric mean of the concentrations for three tetraspanins was calculated to better reflect the total yield of NDEV biomarkers for each sample since it is now well-established that different sub-populations of EVs exist with variable tetraspanin expression [27,28]. Essentially, normalization of NDEV biomarkers by the geometric mean of tetraspanins is used to express their levels per standardized unit of EV content for each sample (conceptually equivalent to expressing the measured concentration of each biomarker per recovered EV).

### 2.5. Statistical Analysis

To account for varying EV isolation efficiency, NDEV biomarkers were standardized by dividing their values by the geometric mean of their respective canonical EV marker levels (tetraspanins CD9, CD181, and CD63).

To determine if it is possible to discriminate between “pure” AD (*N* = 21) and those with mixed pathologies including AD (*N* = 40), we fit a series of independent sample *t*-tests using individuals’ last biomarker sample. To examine the relationships between antemortem cognitive performance and NDEV biomarkers, we fit linear regressions with a random intercept to account for autocorrelation among individuals contributing more than one sample. All hypothesis tests were two-sided; due to the exploratory nature of our work, we did not adjust *p*-values for multiple comparisons.

## 3. Results

### 3.1. Participants Characteristics

We analyzed 76 plasma samples from 61 participants who underwent autopsy, i.e., 15 participants had two samples available, while 46 had one sample available. The cohort consisted of 31 females and 30 males, 77.2 (9.6) (mean, (SD)) years old. Mean cognitive score (MMSE/MOCA) at last visit was 21.1 (SD = 5.7). A mean of 52.8 months (SD = 27) elapsed between the last ADRC visit with a plasma sample and death. During the pathological examination, different proteinopathies (Lewy Body Disease, FTDL, etc.) and vascular pathologies were observed in addition to AD pathologies (Table 1). Participants were divided into groups of “pure” AD (N = 21) or AD with various degrees of additional pathologies (N = 40) (mild or severe, as per McAleese et al. [29]). 

### 3.2. NDEV A/T/N Biomarkers Did Not Distinguish between Pure AD and AD with Mixed Pathologies

Examined A/T/N biomarkers did not distinguish between individuals with pure AD pathology and those with additional pathologies (for all group comparisons, *p* > 0.05). Moreover, there were no differences in NDEV average diameter and concentration by NTA. Furthermore, individual tetraspanin levels in NDEVs and their geometric means did not differ between groups (*p* > 0.05), ruling out the possibility of differences in the concentration of circulating NDEVs between groups and in their recovery by our methods. 

### 3.3. NDEV Biomarkers and Ante-Mortem Cognitive Performance

**Figure 2:**
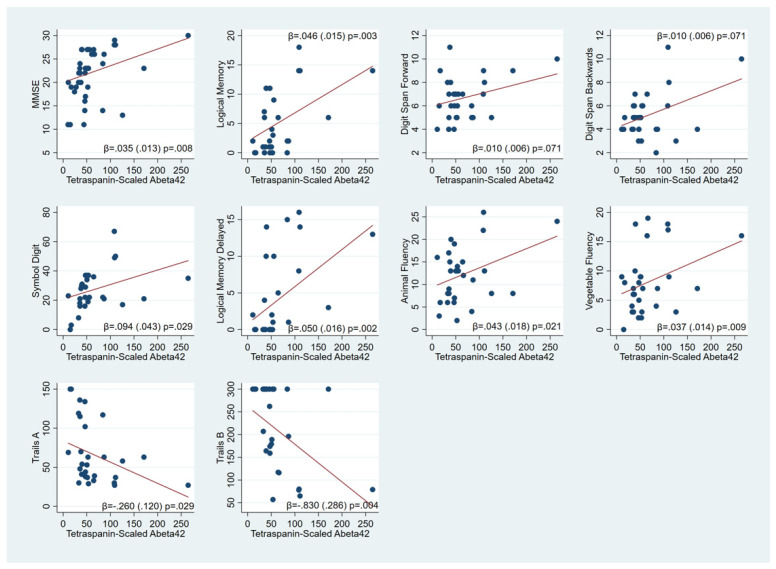
Associations of cognitive scores with NDEV tetraspanin-scaled Aβ_42_ levels (each dot represents a single measured sample for each participant; however, the fit line is derived from the linear regression utilizing all available measurements).

We examined the association of A/T/N biomarkers in NDEVs with different ante-mortem cognitive scores of participants. Higher Aβ_42_ levels were consistently significantly associated with multiple cognitive performance measures indicating better global cognition, memory, fluency, working memory and executive function (Figure 2). In an exploratory fashion, we examined the association between Aβ_42_ levels in NDEVs and Braak staging and found an inverse association trend (β: −0.013; 95% CI −0.026–0.001; *p*: 0.073). Higher NDEV p181-Tau levels were associated with worse digit span backward performance (β: 0.029; 95% CI: 0.002–0.056; *p*: 0.038) and worse TMT-B scores (β: −1.512; 95% CI −3.00–0.028; *p*: 0.046) (Supplementary Figure S1). However, NDEV t-Tau was not associated with ante-mortem cognitive performance (Supplementary Figure S2). Both NDEV t-Tau/Aβ_42_ (β: 0.035; 95% CI: 0.008–0.062; *p*: 0.009) and p-Tau/Aβ_42_ ratios (β: 1.454; 95% CI: 0.676–2.232; *p* < 0.001) were associated with better forward digit span, but p-Tau/Aβ_42_ was associated with worse backward digit span (β: −1.555; 95% CI: −1.984–1.126; *p* < 0.001) (Supplementary Figures S3 and S4). 

**Figure 3:**
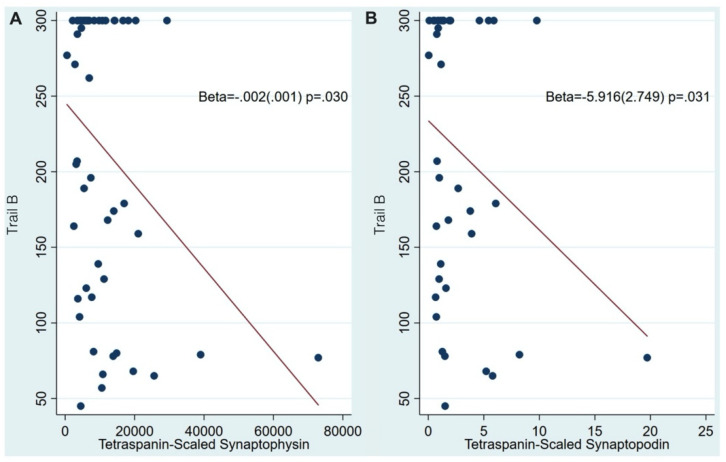
NDEV tetraspanin-scaled synaptopodin (**A**) and synaptopodin (**B**) levels are associated with Trails B scores.

Regarding synaptic integrity-related NDEV markers, higher synaptophysin levels were associated with better digit-span backwards scores (β: 0.00004; 95% CI: 0.00006–0.00002; *p*: 0.028), lower (better) TMT-B scores (β: −0.002; 95% CI: −0.003–−0.001; *p*: 0.030), and (at trend level) with higher (better) animal fluency scores (0.05 < *p* < 0.1). On the other hand, higher synaptopodin levels were only associated with lower (better) TMT-B scores (β: −5.916; 95% CI: −8.665–−3.167; *p*: 0.031) (Figure 3 and Supplementary Figures S5 and S6).

## 4. Discussion

Several lines of evidence suggest that EVs carry aggregation-prone peptides involved in neurodegenerative diseases, including various Aβ and Tau species [30]. We and others have shown that NDEV A/T/N biomarkers, especially p181-Tau and p231-Tau, predict future AD diagnosis and cognitive decline in older people [14,15,31]. Furthermore, in a study involving multiple AD mouse models, we demonstrated that NDEV levels of Aβ_42_ and p181-Tau have moderate to strong correlations with their respective brain levels [32]. In this study, we examined the ability of plasma NDEV biomarkers reflecting A/T/N processes to distinguish between individuals with pure AD pathology and those with AD pathology and various degrees of additional pathologies on autopsy. Moreover, we assessed their ability to track the cognitive severity of clinical AD. The lack of significant differences between individuals harboring only AD pathology and those harboring additional pathologies suggest that these additional pathologies may not modify NDEV A/T/N cargo although biomarkers capturing these additional pathologies might, suggesting an additive effect to that of Aβ and Tau.

Our strongest findings concern NDEV Aβ_42_, which was consistently associated with performance on multiple cognitive domains (cognitive status, memory, fluency, working memory and executive function). In terms of its origin, NDEV Aβ_42_ may reflect intraneuronal endosomal Aβ_42_ [31], according to the mechanism of EV biogenesis. However, the fact that it is largely located on the outer surface of EVs [33] suggests that NDEV Aβ_42_ may also reflect soluble Aβ_42_ that became associated with EVs in the extracellular environment or even in plasma. CSF Aβ_42_ levels in healthy older adults have been associated with future decreases in cognitive performances in [34], which is a report that agrees with our finding of higher NDEV Aβ_42_ levels being associated with better cognitive performance.

NDEV p181-Tau levels were associated with the frontal lobe-mediated executive (Digit Span Backward and TMT-B), although t-Tau was not associated with cognitive variables. Higher CSF tau and p-Tau levels have been associated with worse cognitive performance at the AD dementia stage [35,36], but in our sample blood, NDEV p181-Tau was associated with better performance on Digit Span Backward and Trails B. A benefit of measuring AD-related biomarkers in NDEVs compared to plasma is improved overall detectability, especially in controls. For instance, in this study, NDEV P-tau181 concentrations measured were above the lowest limit of quantification (LLOQ) for all samples, whereas in studies of plasma using even highly sensitive novel assays (P-tau181 Eli Lilly, P-tau181 ADx, and P-tau217 Eli Lilly), a large proportion of control samples were measured below the LLOQs of the assays [37]. Finally, NDEV average diameter, concentration and canonical cargo did not differ across stages of clinical AD, allowing the use of relevant measures as normalizers and arguing against the possibility of changes in the concentration and size of circulating NDEVs induced by the presence of AD pathology. 

Synaptic function underlies cognitive and memory performance and the progressive degeneration of synapses may underly the progressive cognitive decline in AD. Several lines of evidence suggest that synaptic degeneration in AD is a downstream effect of neuroinflammation, tauopathy and amyloidosis [38,39,40,41]. A reduction in the synaptic density in the frontal and temporal cortices, as well as the hippocampus, was documented in autopsy studies of AD brains [42,43,44]. Moreover, synaptic integrity-related protein biomarkers were shown to strongly correlate with cognitive deficits and decline in MMSE scores [45]. Furthermore, lower levels of synaptic proteins were associated with decreased cognitive functions in 4.5-month-old APP/PS1 mice [46]. In contrast with these findings, in the present study, we found that lower levels of synaptic proteins were associated with greater impairments in some frontal lobe-mediated cognitive functions. This suggests that the NDEV cargo of (at least) synaptophysin and synaptopodin may not reflect synaptic damage and degeneration, but rather normal synaptic function, which diminishes with AD progression (as shown for some synaptic proteins [47]). Longitudinal studies spanning preclinical and clinical stages of AD and involving the quantification of functionally distinct synaptic proteins may clarify the relative role of degenerative and normal synaptic processes in EV loading with synaptic cargo and might resolve such apparent contradictions. 

This study is remarkable in that, to the best of our knowledge, it is the first examination of NDEV biomarkers in autopsy-confirmed AD. NDEV biomarkers offer both theoretical and practical benefits compared to plasma biomarkers of AD, which typically show low blood concentrations against a complex matrix that further limits their detectability. Conceptually, the attribution of plasma biomarkers to brain pathology is challenged by the fact that peripheral tissues may also produce the marker of interest and may deliver it to the circulation without the need to cross a complex barrier, such as the blood–brain barrier. The (largely) neuronal derivation of L1CAM + EVs addresses this conceptual challenge and brings us closer to a brain “liquid biopsy”. Finally, whereas multiple candidate plasma biomarkers have failed to reproduce key findings other than their original cohorts, this is not the case for NDEV biomarkers, since original findings [13,48] were reproduced blindly by us [15] and others [17], and their biological plausibility was demonstrated in multiple animal models [32]. Limitations include the lack of an autopsy-confirmed control group, which would have allowed us to examine differences between AD and control participants. Due to limited sample availability and the nature of our methodology, which isolates a subset of total circulating EVs, our ability to measure multiple protein biomarkers was limited. Although in principle desirable, we did not prioritize measurement of Nfl in NDEVs from this cohort, choosing to favor Aβ-, Tau- and synaptic integrity-related biomarkers. One factor for this decision is the fact that Nfl in other biofluids appears to be a non-specific biomarker of neuronal damage. For instance, in a recent study, plasma NfL showed associations with cognition and imaging markers of neurodegeneration, but, in contrast to plasma p181-Tau, these associations were not AD specific [49]. Limited amounts of available stored plasma also prevented us from examining a wider range of NDEV biomarkers that might have allowed us to discover biomarkers distinguishing between pure AD and AD with mixed pathologies. Moreover, the predominance of AD pathology in all examined cases and the lack of cases with pure vascular dementia and/or LBD discouraged us from investigating NDEV biomarkers that could be more specifically tied to these specific pathologies (e.g., a-synuclein, TDP43, vascular endothelial proteins). Notably, early pathologic stages of AD by Braak were relatively under-represented in this cohort. Future research should aim to expand upon our findings and circumvent these difficulties. The ongoing development of biomarkers from EVs reflecting other brain cell types, i.e., astrocytic [50,51] and/or endothelial EVs [52] may supplement A/T/N biomarkers and expand our conceptual framework for approaching cases of AD mixed with other pathologies along the lines of Precision Medicine and ultimately allow clinicians to tailor secondary prevention and clinical treatment strategies depending on the underlying pathology.

## Figures and Tables

**Table 1:** Demographics of the participants for this study.

Variables	Pure AD (*n* = 21)	Mixed AD (*n* = 40)	Total (*n* = 61)
Age at time of blood draw, mean (SD)	76.9 (12.3)	77.3 (8.5)	77.2 (9.6)
Male	13 (62%)	17 (43%)	30 (49%)
Race [White (%)/African American (%)]	21 (100%)/0 (0%)	35 (88%)/5 (13%)	56 (92%)/5 (8%)
Education, mean (SD)	15.7 (2.9)	15.7 (2.8)	15.7 (2.8)
MMSE total, mean (SD)	21.3 (5.9)	21.0 (5.6)	21.1 (5.7)
Years of follow-up from (earliest, if more than one) sample to death, mean (SD)	8.6 (8.3)	7.3 (3.7)	7.7 (5.7)
Months from Visit to Death (mean (SD))	50.7 (24.5)	53.7 (28.2)	52.8 (27.0)

## Data Availability

The data presented in this study are available on request from the corresponding author.

## Supplementary Materials

The following are available online at https://www.mdpi.com/article/10.3390/cells11030436/s1, Figure S1: Associations of cognitive scores with NDEV tetraspanin-scaled p181-Tau levels, Figure S2: Associations of cognitive scores with NDEV tetraspanin-scaled total Tau levels, Figure S3: Associations of cognitive scores with NDEV tetraspanin-scaled p181-Tau/Aβ42 Ratios, Figure S4: Associations of cognitive scores with NDEV tetraspanin-scaled total Tau/Aβ42 Ratios, Figure S5: Associations of cognitive scores with NDEV tetraspanin-scaled Synaptophysin levels, Figure S6: Associations of cognitive scores with NDEV tetraspanin-scaled Synaptopodin levels.
