# Supplementary material for: Neuronal-Derived EV Biomarkers Track Cognitive Decline in Alzheimer’s Disease
Source: Cells. 2022 Jan 27;11(3):436. doi: 10.3390/cells11030436 (PMC8834433; doi:10.3390/cells11030436)

Supplementary Figure S1. Associations of cognitive scores with NDEV tetraspanin-scaled p181-Tau levels

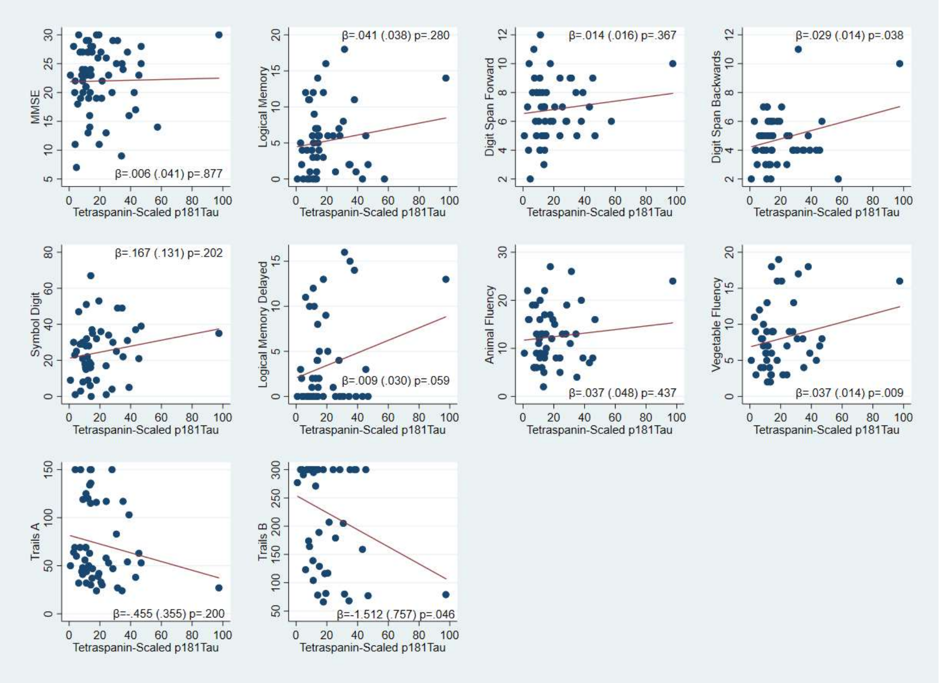

Supplementary Figure S2. Associations of cognitive scores with NDEV tetraspanin-scaled total Tau levels

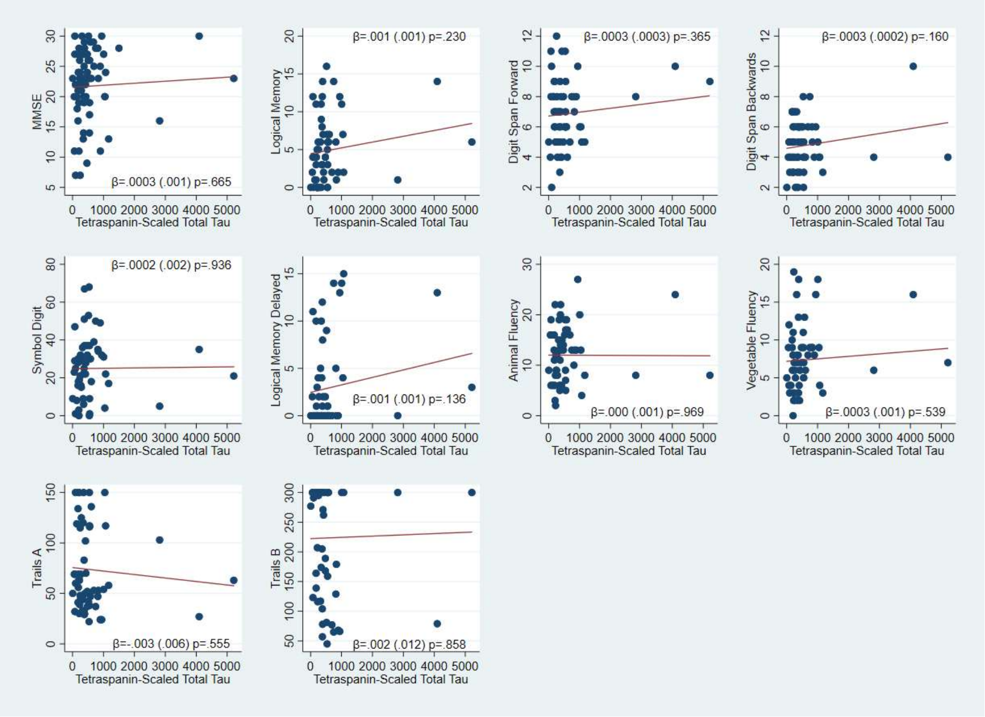

Supplementary Figure S3. Associations of cognitive scores with NDEV tetraspanin-scaled p181-Tau/Aβ_{42} Ratios

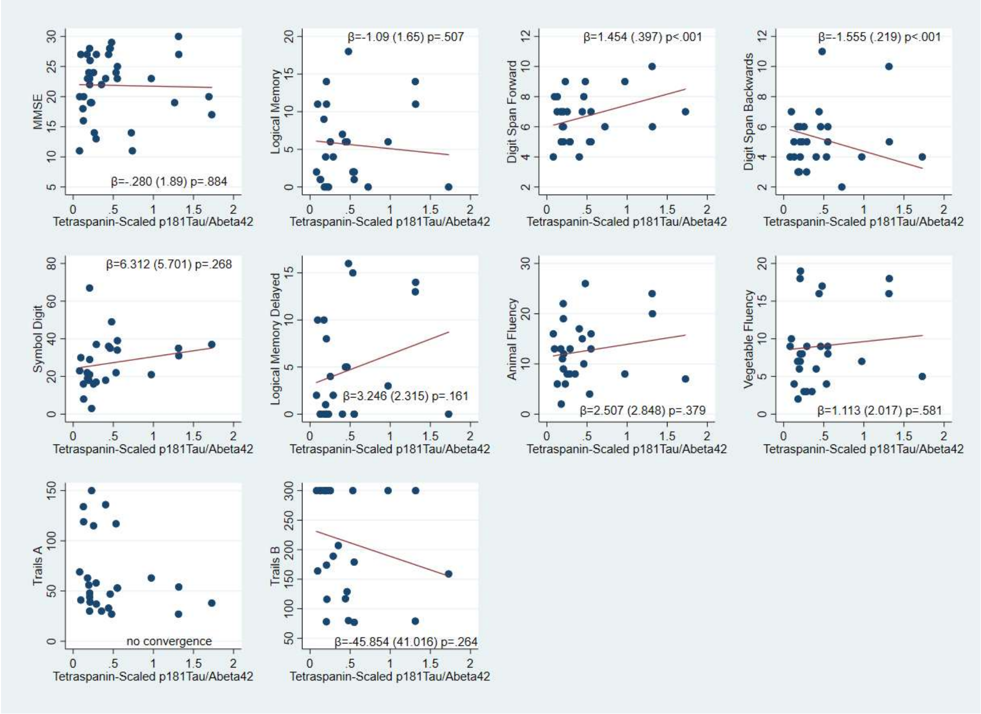

Supplementary Figure S4. Associations of cognitive scores with NDEV tetraspanin-scaled total Tau/Aβ_{42} Ratios

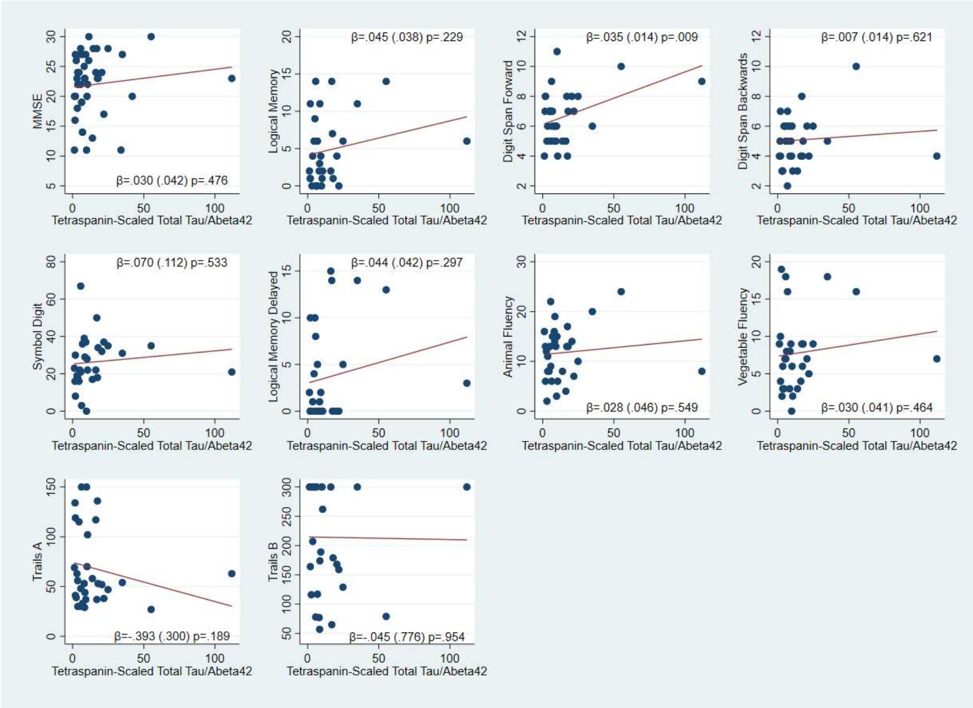

Supplementary Figure S5. Associations of cognitive scores with NDEV tetraspanin-scaled Synaptophysin levels

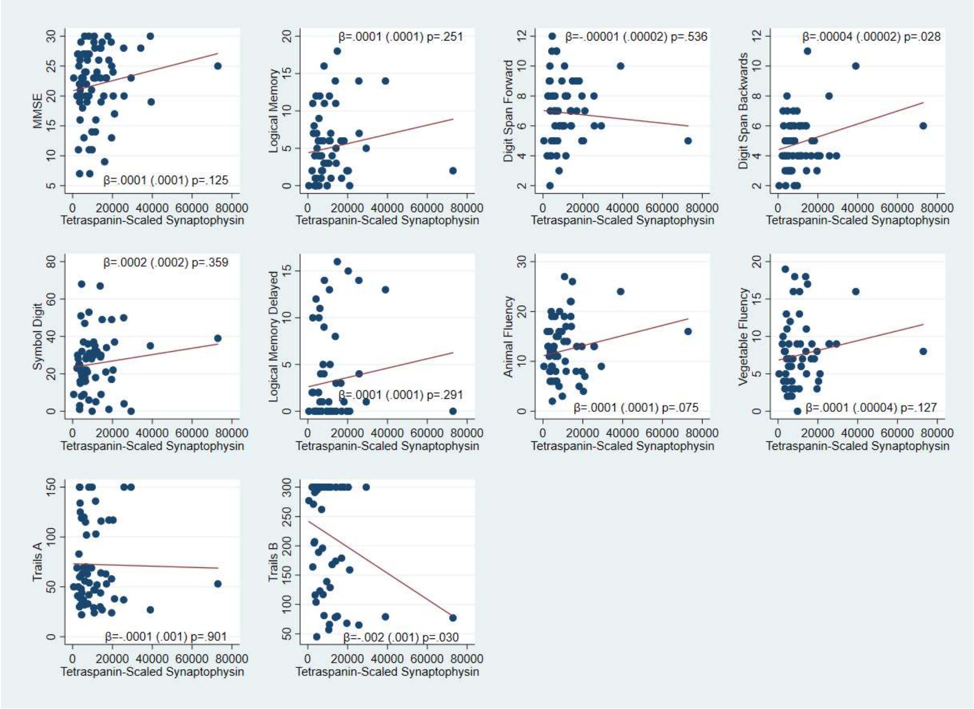

Supplementary Figure S6. Associations of cognitive scores with NDEV tetraspanin-scaled Synaptopodin levels

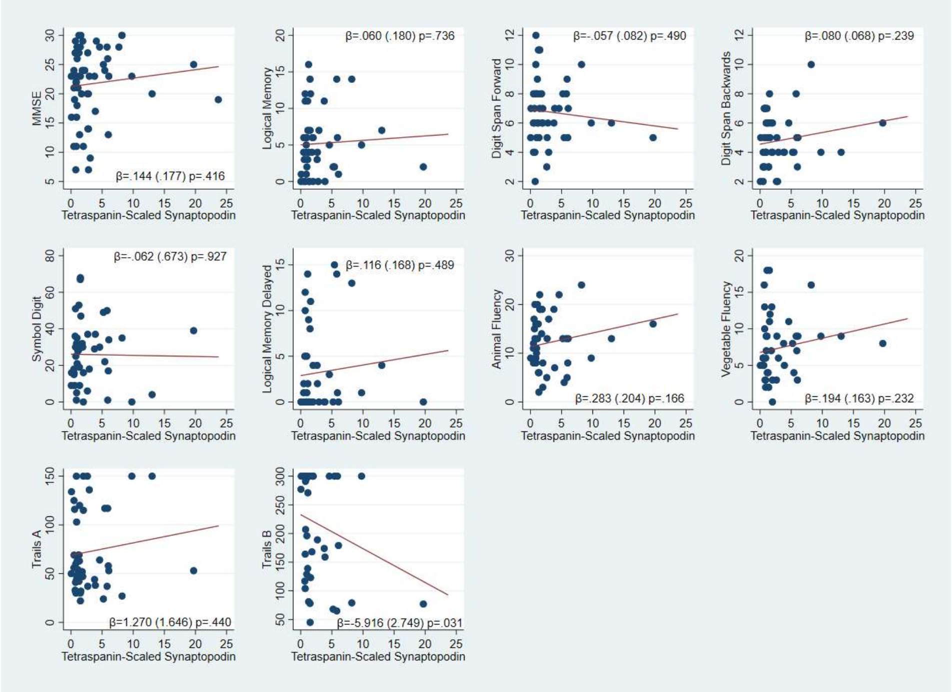

Supplement: Supplementary file 1 [file cells-11-00436-s001.zip › cells-1520708-supplementary.pdf]
